# The Biological and Molecular Function of LINC00665 in Human Cancers

**DOI:** 10.3389/fonc.2022.886034

**Published:** 2022-05-19

**Authors:** Cheng Zhang, Shu-Ning Xu, Ke Li, Jing-Hong Chen, Qun Li, Ying Liu

**Affiliations:** Department of Oncology, The Affiliated Cancer Hospital of Zhengzhou University and Henan Cancer Hospital, Zhengzhou, China

**Keywords:** cancer, lncRNA, LINC00665, biological roles, biomarker

## Abstract

Long non-coding RNAs (lncRNAs) are more than 200 nucleotides in length and are implicated in the development of human cancers, without protein-coding function. Mounting evidence indicates that cancer initiation and progression are triggered by lncRNA dysregulation. Recently, a growing number of studies have found that LINC00665, a long intergenic non-protein coding RNA, may be associated with various cancers, including gastrointestinal tumors, gynecological tumors, and respiratory neoplasms. LINC00665 was reported to be significantly dysregulated in cancers and has an important clinical association. It participates in cell proliferation, migration, invasion, and apoptosis through different biological pathways. In this review, we summarize the current findings on LINC00665, including its biological roles and molecular mechanisms in various cancers. LINC00665 may be a potential prognostic biomarker and novel therapeutic target for cancers.

## Introduction

With an increase in aging and population growth, the incidence and mortality of human cancers are growing rapidly worldwide (1). According to statistical estimations from 185 countries, there were more than 18 million new cancer cases and almost 10 million cancer-related deaths in 2018 (1). Although numerous treatment strategies for cancers have been developed and improved, including classical therapies (surgery, radiotherapy, and chemotherapy) and immune and molecular targeted therapies, the therapeutic effects remain unsatisfactory (2, 3). Thus, there is an urgent need to explore valuable targets and novel biomarkers for cancer therapy, diagnosis, and prognostic evaluation.

Long non-coding RNAs (lncRNAs), a class of linear RNA molecules with a length greater than 200 nt, have no protein-coding ability, and their average expression and abundance are lower than that of mRNA (4, 5). Mounting studies have found that lncRNAs were dysregulated in human diseases, especially in cancers, and could act as mediators in tumorigenesis and metastasis. LncRNAs have a wide subcellular distribution in cells, which determines the diversity of their functional mechanisms (6, 7). For example, lncRNAs located in the cytoplasm can regulate mRNA stability as competing endogenous RNAs (ceRNAs) through sponging specific miRNAs (8–10). In the nucleus, the lncRNA PVT1 could increase MYC stability and its expression level in cancers by interfering with the phosphorylation of MYC at Thr58 (11). Furthermore, emerging evidence has shown that lncRNAs might be valuable diagnostic and prognostic biomarkers or therapeutic targets for cancers (12–15). Although lots of lncRNAs have been identified in cancers, the biological function of several remains unclear.

Long intergenic non-protein coding RNA 665 (LINC00665), also known as CIP2A-BP, is a novel lncRNA that is dysregulated in different human cancers. Recent studies have found that LINC00665 expression was significantly upregulated in most cancers and could be used as a valuable diagnostic, prognostic, and therapeutic target. LINC00665 plays an oncogenic role in cancer cell proliferation, migration, and invasion through various molecular mechanisms. All these findings indicate that LINC00665 has a key function in cancer. The present review summarizes current findings of LINC00665 in tumorigenesis and progression, including aberrant expression, clinical value, and molecular mechanism (**Tables 1**, **2**).

**Table 1:** Biological characterization of LINC00665 in human cancers.

Cancer types	Expression	Subcellular localization	Targets	Biological function	References
Breast cancer	Up	Cytoplasm	miR-379-5p-LIN28B, miR-3619-5p/CTNNB1, miR-551b-5p, encodes micropeptide CIP2A-BP	Proliferation, migration, invasion, apoptosis, tumor growth, and EMT process	(16–21)
Prostate cancer	Up	Nuclear	Inhibit KLF2 transcription *via* recruiting EZH2, LSD1, and miR-329-RBBP8/XPC	Proliferation, migration, invasion, tumor growth, and radiation-induced residual DNA damage	(22–24)
Hepatocellular carcinoma	Up	NA	LINC00665-PKR-NF-κB feedback loop, miR-186-5p-MAP4K3, CDK1, BUB1B, BUB1, PLK1, CCNB2, CCNB1, CDC20, ESPL1, MAD2L1, and CCNA2	Proliferation, cell cycle, apoptosis, autophagy, and tumor growth	(25–27)
Osteosarcoma	Up	Cytoplasm	miR-3619-5p, miR-708, and miR-142-5p-RAP1B	Proliferation, migration, and invasion	(28, 29)
Colorectal cancer	Up	Cytoplasm	miR-9-5p-ATF1, miR-214-3p-CTNNB1/Wnt/β-catenin signaling pathway, miR-126-5p-PAK2/FZD3	Proliferation, migration, invasion, and apoptosis	(30–32)
Gastric cancer	Up	NA	miR-149-3p-RNF2, Wnt signaling pathway, TGF-β/Smad pathway, miR-379-5p-GRP78	Proliferation, migration, invasion, apoptosis, and tumor growth	(33–36)
Glioma	Up/Down	Cytoplasm	TAF15/LINC00665/MTF1(YY2)/GTSE1 axis, miR-34a-5p-AGTR1	Proliferation, migration, invasion, apoptosis, and tumor growth	(37, 38)
Lung cancer	Up	Cytoplasm	miR-138-5p-E2F3, EZH2-CDKN1C, recruiting EZH2 and activating PI3K/AKT pathway, YB-1-ANGPT4/ANGPTL3/VEGFA axis, miR-98-AKR1B10-ERK signaling pathway, miR-181c-5p-ZIC2 axis, miR-195-5p-MYCBP, miR-let-7b-5p-CCNA2	Proliferation, migration, invasion, apoptosis and tumor growth, drug resistance/sensitivity	(39–46)
Melanoma	Up	NA	miR-224-5p-VMA21	Proliferation and migration	(47)
Endometrial carcinoma	UP	NA	Co-immunoprecipitated with HMGA1 protein	Proliferation, migration, invasion, apoptosis, and tumor growth	(48)
Cholangiocarcinoma	Up	NA	miR-424-5p-BCL9L	Cell sphere formation, migration, and invasion	(49)
Ovarian cancer	Up	Cytoplasm	miRNA-34a-5p-E2F3, miR-449b-5p-VAV3/RRAGD, miR-146a-5p-CXCR4	Proliferation, migration, invasion, lymphocyte infiltration, and autophagy	(50–53)
Leukemia	Up	NA	miR-4458-DOCK1, miR-101-PI3K/Akt pathway	Proliferation, migration, invasion, and apoptosis	(54, 55)
Cervical cancer	NA	NA	WNT-CTNNB1/β−catenin signaling pathway	Proliferation, migration, invasion, and EMT	(56)

NA, Not available.

**Table 2:** Clinical features of LINC00665 in human cancers.

Cancer types	Clinical features of LINC00665	References
Breast cancer	Tumor size, TNM stage, lymph node metastasis, postoperative pathological lymph node status, negative predictor for pathological complete response, and poor prognosis	(16–21)
Prostate cancer	T stage, lymph node metastasis, radiation resistance, and poor prognosis	(22–24)
Hepatocellular carcinoma	Tumor differentiation grade, TNM stage, tumor size, Edmondson grade, and poor prognosis	(25–27)
Osteosarcoma	Tumor size, clinical stages, and poor prognosis	(28, 29)
Colorectal cancer	Local lymph node metastasis and poor differentiation	(30–32)
Gastric cancer	TNM stage, tumor depth, lymph node metastasis, histological grade, poor prognosis, and cisplatin resistant	(33–36)
Glioma	Clinical stage, poor prognosis, and negatively correlated with pathological grade	(37, 38)
Lung cancer	Lymph node metastasis, TNM stage, lymph node metastasis, tumor size, gefitinib resistance, and poor prognosis	(39–46)
Cholangiocarcinoma	TNM stage, lymph node metastasis, distant metastasis, poor prognosis, and gemcitabine chemoresistance	(49)
Ovarian cancer	Tumor size, FIGO stage, lymph node metastasis, and poor prognosis	(50–53)

## Characterization of LINC00665

**Figure 1:**
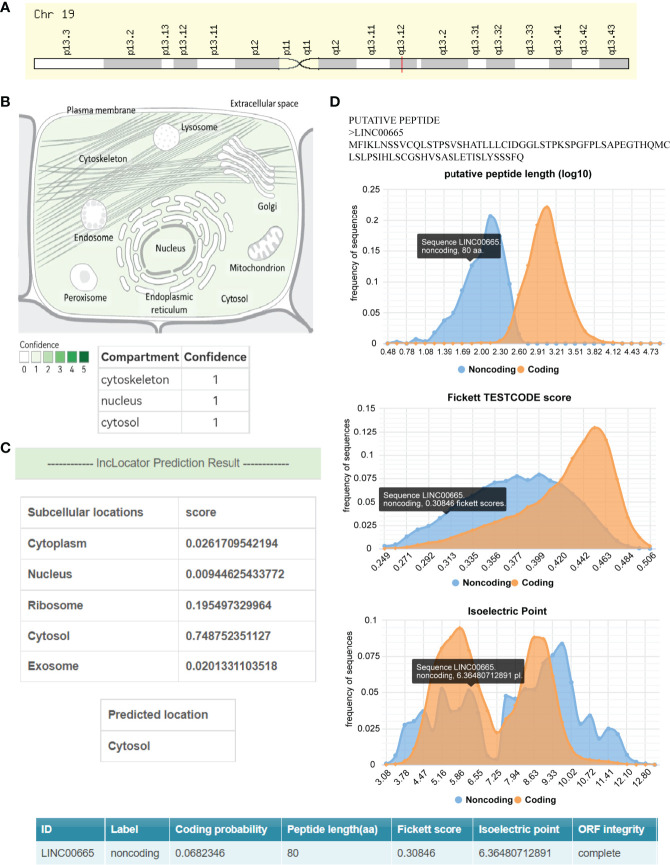
The subcellular localization and coding potential of LINC00665. **(A)** Chromosome 19q13.12 has a cytogenetic band for LINC00665. **(B)** LINC00665 expression concentrates in the nucleus, cytosol, and cytoskeleton. **(C)** The subcellular localization of LINC00665 predicted by lncLocator was the cytoplasm. **(D)** The protein-coding probability features of LINC00665, including Fickett score, peptide length (synonymous with ORF length), and isoelectric point.

**Figure 2:**
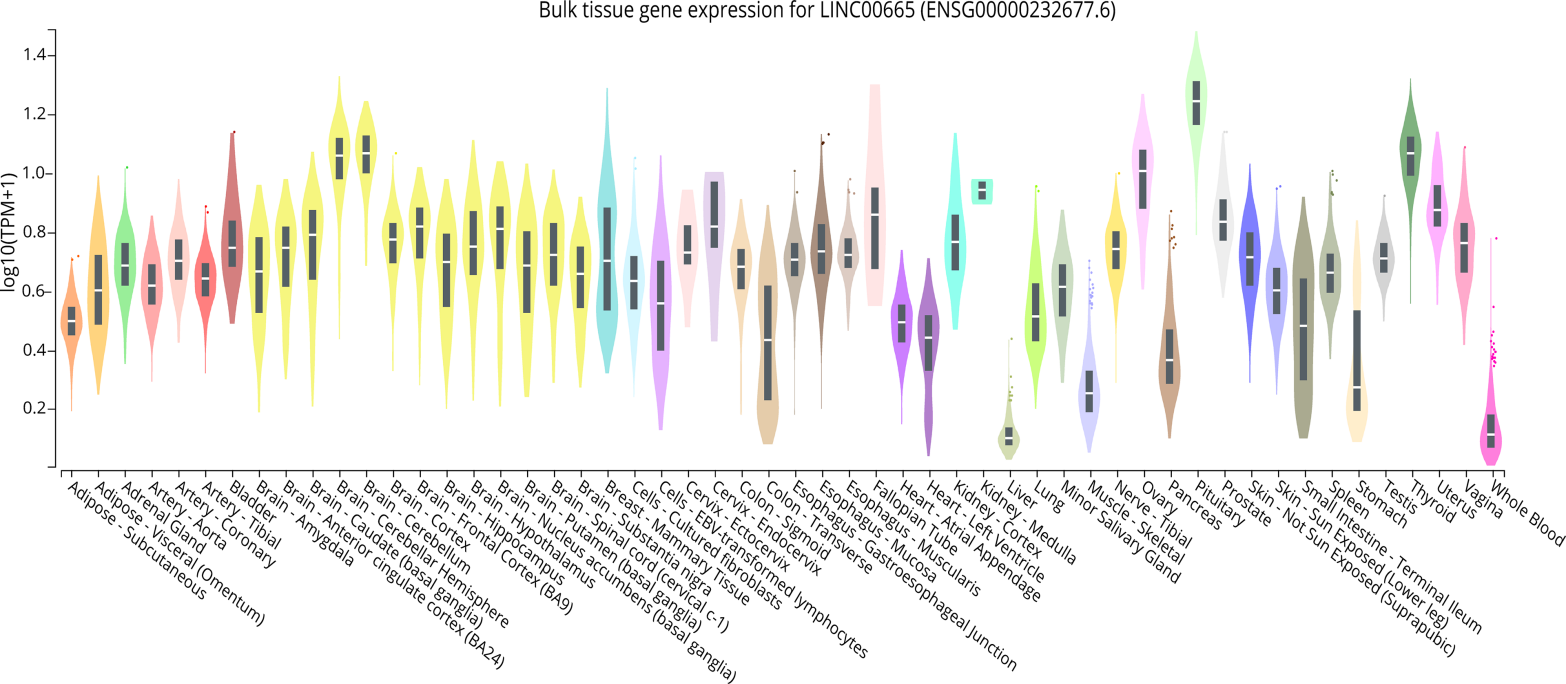
LINC00665 expression in various kinds of human normal tissues (data from GTEx).

**Figure 3:**
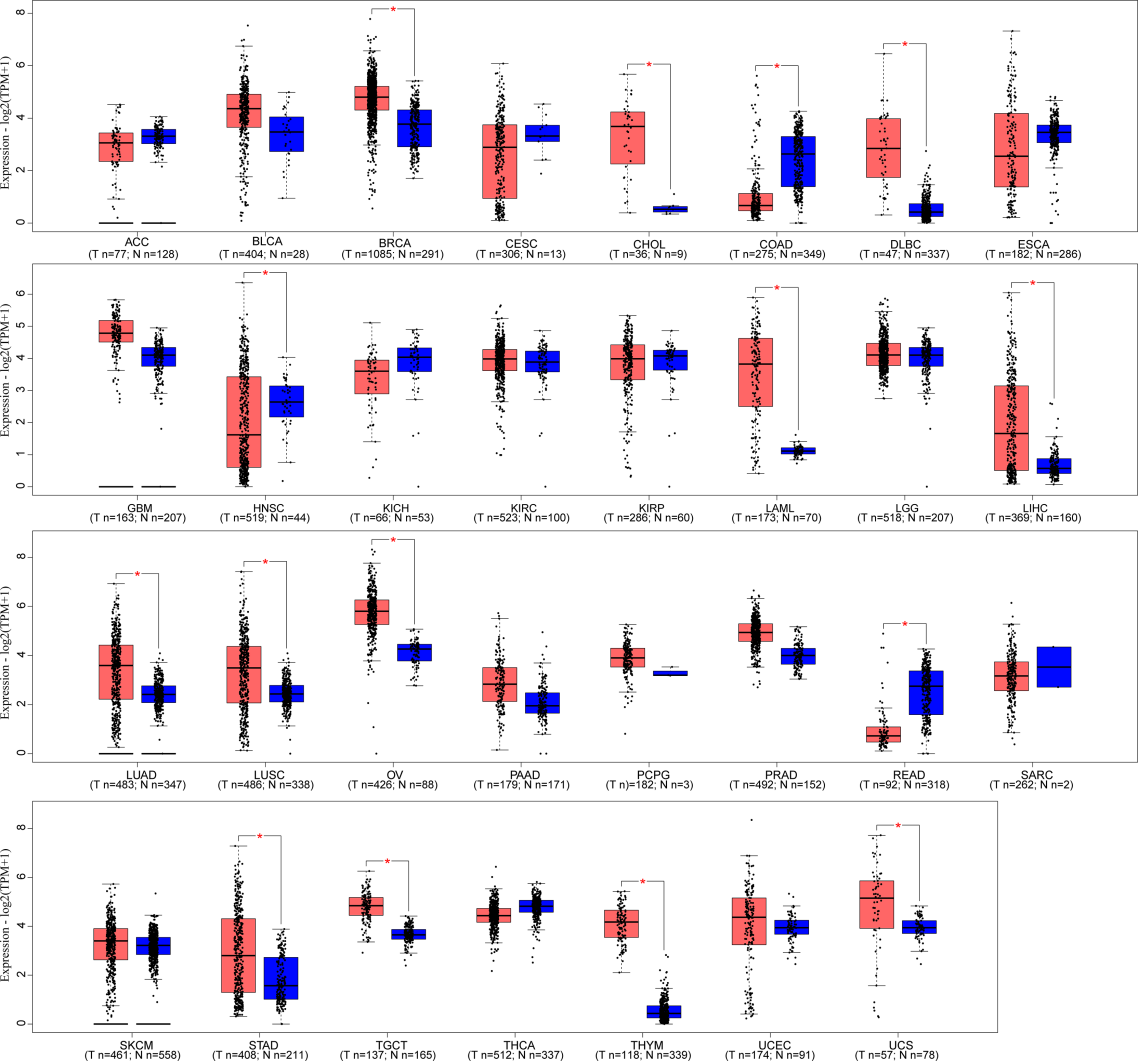
LINC00665 expression in human cancers and corresponding normal tissues (data from TCGA and GTEx). *p < 0.05.

**Figure 4:**
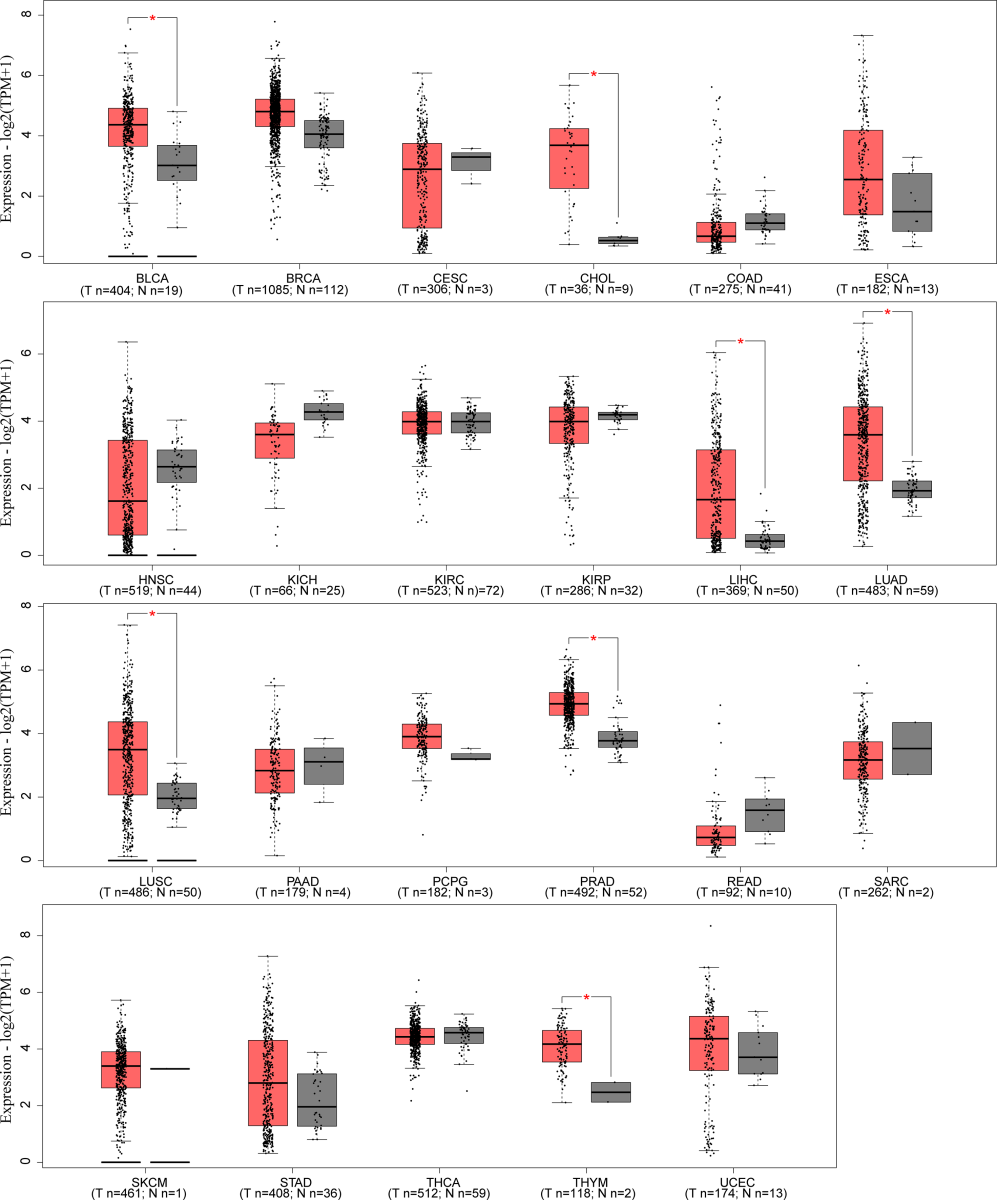
LINC00665 expression in human cancers and corresponding normal tissues (data from TCGA). *p < 0.05.

LINC00665,on chromosome19q13.12, is a RNA gene with a length of 1,749 bp containing eight exons (**Figure 1A**). First, we explored the subcellular localization of LINC00665 using the COMPARTMENTS tools (57) and it showed that LINC00665 was expressed in the nucleus, cytosol, and cytoskeleton (**Figure 1B**). The lncLocator (58) predicted that the score of LINC00665 located in the cytosol was the highest (**Figure 1C**). CPC algorithm V2.0 (59) was used to calculate the coding potential of LINC00665. As shown in **Figure 1D**, LINC00665 was predicted as a non-coding transcript. The expression levels of LINC00665 in human cancers and normal tissues were explored using the Genotype-Tissue Expression (GTEx) Project (60) and Gene Expression Profiling Interactive Analysis (GEPIA) (61). It was found that LINC00665 was broadly expressed in different human tissues (**Figure 2**). The data from TCGA and GTEx revealed that LINC00665 was significantly differentially expressed in breast invasive carcinoma, cholangiocarcinoma, colon adenocarcinoma, lymphoid neoplasm diffuse large B-cell lymphoma, head and neck squamous cell carcinoma, acute myeloid leukemia, liver hepatocellular carcinoma, lung adenocarcinoma, lung squamous cell carcinoma, ovarian serous cystadenocarcinoma, rectum adenocarcinoma, stomach adenocarcinoma, testicular germ cell tumors, thymoma, and uterine carcinosarcoma (**Figure 3**). The expression of LINC00665 in cancer and normal tissues analyzed using TCGA data is shown in **Figure 4**.

## LINC00665 Dysregulation and Biological Roles in Human Cancers

### LINC00665 in Breast Cancer

The subcellular localization of LINC00665 in BCa cells is well studied. Evidence based on fluorescence *in situ* hybridization (FISH) and subcellular fractionation followed by RT-qPCR showed that LINC00665 was mainly located in the cytoplasm (16, 17). LINC00665 is remarkably elevated in 106 BCa tissues and cell lines (MDA-MB-231 and MCF-7), and its expression is significantly associated with tumor size and TNM stage. LINC00665 knockdown suppresses BCa cell proliferation, migration, and invasion, but promotes cell apoptosis. Mechanically, LINC00665 silencing could inhibit β-catenin expression through competitively binding miR-3619-5p in BCa cells (18). Increasing evidence found that LINC00665 acted as a sponge for miR-379-5p (16) and miR-551b-5p (19) in BCa. Dai et al. (20) explored the correlation between LINC00665 expression and pathological complete response (pCR) in 102 neoadjuvant chemotherapy BCa patients. The results showed that LINC00665 expression was an independent predictor of pCR (OR = 0.351, 95% CI: 0.125–0.936, *p* = 0.040), especially in patients with hormone receptor-positive/human epidermal growth factor receptor 2-negative subtypes (OR = 0.272, 95% CI: 0.104–0.664, *p* = 0.005). Although LINC00665 has no protein-coding function, it was found to encode biologically active micropeptide. Guo et al. (17) revealed that LINC00665 was regulated by TGF-β at the translational level and encoded micropeptide CIP2A‐BP, thus participating in BCa progression. LINC00665 may promote BCa cell metastasis by triggering the epithelial–mesenchymal transition (EMT) process (21).

### LINC00665 in Prostate Cancer

Xue et al. (22) observed that LINC00665 expression was upregulated in 50 PCa tissues and cell lines (LNCaP, 22RV1, PC-3,and DU-145) compared with corresponding control groups. Upregulated LINC00665 expression was correlated with the advanced T stage, lymph node metastasis, and the poor survival of PCa patients. Moreover, LINC00665 knockdown significantly suppressed PCa cell proliferation and migration. To further explore the biological mechanism of LINC00665, the subcellular fractionation assay found that LINC00665 was mainly located in the nucleus of PCa (22RV1 and DU-145 cells). Mechanically, LINC00665 may epigenetically inhibit KLF2 transcription and expression through recruiting EZH2 and LSD1 to its promoter region, thus playing an oncogenic role in PCa. LINC00665 could also promote PCa progression as a ceRNA through the miR-1224-5p/SND1 axis (23). Eke et al. (24) studied radiation-induced lncRNA dysregulation and found that LINC00665 was upregulated in LNCaP PCa cells at 2 months after a single dose of 10 Gy but not after multifractionated irradiation. Subsequent cell function assays showed that LINC00665 regulated the expression of the DNA repair proteins CtIP (RBBP8) and XPC.

### LINC00665 in Hepatocellular Carcinoma

Compared with adjacent normal tissues and a normal liver cell line HL-7702, LINC00665 was highly expressed in 76 HCC tissues and HCC cell lines (Huh-7, HepG2, HCCLM6, MHCC-97H, and Hep3B) (25). High LINC00665 expression was associated with the advanced tumor size, Edmondson grade, and the poor survival of HCC patients. *In vitro* and *in vivo* assays indicated that LINC00665 knockdown inhibited HCC cell viability and tumor growth, and induced apoptosis and autophagy. Further molecular regulatory investigation revealed that LINC00665 modulated the expression of MAP4K3 by sponging miR-186-5p. The findings of upregulated LINC00665 in HCC were also confirmed by Wen et al. (26) using TCGA, GEO, and quantitative real-time polymerase chain reaction (qRT-PCR) data. Ding et al. (27) performed an RNA-sequencing analysis in Huh-7 cells that were treated with TNFα, IL-1β, or INF-γ to identify the NF-κB-associated lncRNAs in HCC, and found that LINC00665 was the most highly induced upregulated lncRNA. In line with other studies, LINC00665 was upregulated in (84/122) HCC tissues and its expression was positively correlated with TNM stages, Barcelona Clinic Liver Cancer (BCLC) stages, and the poor prognosis of HCC patients. Furthermore, LINC00665 physically interacted with PKR and played an oncogenic role by promoting PKR activation and stability, thus giving feedback on prosperous NF-κB signaling in HCC.

### LINC00665 in Colorectal Cancer

Wu et al. (30) measured the expression level of LINC00665 in 67 pairs of CRC tissues and cell lines by qRT-PCR. The findings showed that LINC00665 was overexpressed in CRC tissues and cell lines, and LINC00665 knockdown suppressed CRC cell proliferation and promoted cell apoptosis. The subcellular distribution of LINC00665 in CRC cells was mainly in the cytoplasm. Mechanistically, LINC00665 could sponge miR-126-5p to regulate PAK2 and FZD3 expression. A study by Han et al. (31) also found that LINC00665 was abnormally upregulated in CRC cells and mainly located in the cytoplasm of HCT-116 and SW480 cells. Molecular investigations found that LINC00665 could increase the expression of CTNNB1 by sponging miR-214-3p or binding to U2AF2 protein, further activating the Wnt/β-catenin signaling pathway to promote the tumorigenesis of CRC. In addition, upregulated LINC00665 stimulated CRC progression by regulating the miR-9-5p/ATF1 axis (32).

### LINC00665 in Gastric Cancer

In the digestive tract, GC remains a common malignant tumor with a high incidence and mortality and a poor prognosis (62). LINC00665 was first identified to be overexpressed in 49 paired GC tissues and cell lines in a study by Qi et al. (33). High LINC00665 expression was also found to be associated with an advanced TNM stage and the histological grade of GC. Moreover, *in vitro* assays showed that LINC00665 may promote the proliferation and invasion of GC cells, and as a ceRNA for miR-149-3p to regulate the expression of RNF2. Yang et al. (34) demonstrated that LINC00665 silencing could significantly inhibit GC cell proliferation, migration, invasion, and induce apoptosis *in vitro*, as well as restrain tumor growth *in vivo*. The Wnt pathway, also named Wnt/β-catenin, was known to play a crucial role in GC tumorigenesis and was found to be activated by LINC00665. In addition, LINC00665 could facilitate the proliferation, invasion, and metastasis of GC cells *via* activating the TGF-β signal pathway (35). Acquired resistance to cisplatin (DDP)-based chemotherapy is a common clinical issue in the treatment of GC. Yue et al. (36) reported that LINC00665 expression was higher in DDP-resistant GC cell lines than that in normal gastric mucosal epithelial cell lines and DDP-sensitive GC cell lines. LINC00665 knockdown inhibited DDP-resistant GC cell proliferation, induced apoptosis, and improved its DDP sensitivity by suppressing endoplasmic reticulum (ER) stress.

### LINC00665 in Lung Cancer

According to the pathological pattern, lung cancer cases are divided into non-small cell lung cancer (NSCLC) and small cell lung cancer. NSCLC accounts for approximately 85% and its primary subtype is lung adenocarcinoma. Yang et al. (39) found that LINC00665 was upregulated in lung cancer tissues by analyzing the TCGA database, and further qRT-PCR assay confirmed that it was highly expressed in 51 of 60 NSCLC tissues. High LINC00665 expression was associated with advanced tumor size, TNM stage, and lymph node metastasis. Kaplan–Meier survival analysis indicated that patients with high LINC00665 expression had poorer overall survival (OS) and progression-free survival than those with low expression. Functionally, LINC00665 knockdown suppressed NSCLC cell proliferation and migration and promoted cell apoptosis. Further assays *in vitro* and *in vivo* showed that LINC00665 knockdown improved the sensitivity of NSCLC cells to DDP. Based on the findings that LINC00665 was primarily distributed in the cytoplasm of NSCLC cells, LINC00665 was found to recruit EZH2 to the CDKN1C promoter region to facilitate the demethylation of histone H3K27 and inhibit CDKN1C transcription. As a ceRNA, LINC00665 could regulate the expression of E2F3, AKR1B10, ZIC2, MYCBP, and CCNA2 by sponging miR-138-5p (40), miR-98 (41), miR-181c-5p (42), miR-195-5p (43), and miR-let-7b (44), respectively. LINC00665 also participated in tumor angiogenesis. Cong et al. (45) uncovered that YB-1 protein stability was improved by directly interacting with LINC00665, and thus activated the transcription and expression of ANGPT4, ANGPTL3, and VEGFA by binding to their promoters in the process of tumor angiogenesis. A recent study also found that LINC00665 was overexpressed in lung cancer tissues and cells with acquired gefitinib resistance (46). A series of assays *in vitro* and *in vivo* confirmed that LINC00665 promoted the resistance of NSCLC cells to gefitinib by increasing EZH2 and activating the PI3K/AKT pathway.

### LINC00665 in Ovarian Cancer

OC, with high mortality and poor prognosis, is one of the most common malignant tumors in the female reproductive system. Gao et al. (50) found that LINC00665 was upregulated in OC tissues by analyzing the GEPIA database and constructing a prognosis correlated with the LINC00665-miR-146a-5p-CXCR4 regulatory network. LINC00665 expression was positively correlated with infiltrating levels of CD4+ T cells and negatively correlated with CD8+ T cells, neutrophils, macrophages, and dendritic cells, which demonstrated the correlation between LINC00665 and lymphocyte infiltration in high-grade serous OC (51). Moreover, a nine-autophagy-related lncRNA signature (including LINC00665) was constructed as independent prognostic factors for the OS of OC patients (52). LINC00665 may regulate E2F3 expression through competitively binding to miRNA-34a-5p in promoting OC progression (53).

### LINC00665 in Glioma

Through lncRNA microarray analysis, LINC00665 was identified to be differentially expressed in glioma. Further RT-qPCR assays confirmed the high expression levels of LINC00665 in 48 glioma tissues and cell lines. Based on the evidence that LINC00665 was mainly distributed in the cytoplasm, Dai et al. discovered that the LINC00665/miR-34a-5p/AGTR1 axis contributed to the development and progression of glioma (37). In addition to the ceRNA regulatory pathway, lncRNAs, which were located in the cytoplasm, could regulate mRNA expression through STAU1-mediated mRNA degradation (63). Ruan et al. (38) reported that LINC00665 was downregulated in glioma tissues and cells and acted as a tumor suppressor gene. Further exploration found that LINC00665 facilitated the degradation of MTF1 and YY2 mRNA *via* interacting with a double-stranded RNA-binding protein STAU1. The Alu elements on LINC00665 and MTF1 or YY2 mRNA 3’UTR constructed a STAU1-binding site through complementary base pairing; thus, STAU1 executed mRNA decay *via* binding to the constructed site.

### LINC00665 in Other Cancers

LINC00665 expression was upregulated in 42 osteosarcoma tissues and four cell lines. Further clinicopathological correlation analysis indicated that higher LINC00665 expression was correlated with larger tumor size, later clinical stages, and poorer OS. Mechanistically, LINC00665 facilitated osteosarcoma progression by increasing RAP1B expression *via* targeting miR-708 and miR-142-5p (28). LINC00665 could also promote osteosarcoma progression by sponging miR-3619 (29). Compared with normal human epidermal melanocytes, LINC00665 expression was significantly increased in four melanoma cells lines (A375, M21, A2058, and A-875). A series of assays *in vitro* and *in vivo* showed that LINC00665 was mainly expressed in the cytoplasm and could promote the malignant behaviors of melanoma cells through the miR-224-5p/VMA21 axis (47). Xia et al. (56) demonstrated that LINC00665 may promote HeLa cell proliferation, metastasis, and EMT *via* the WNT-CTNNB1/β−catenin signaling pathway. In endometrial carcinoma, LINC00665 was overexpressed in endometrial carcinoma tissues and cell lines. Mechanistically, LINC00665 co-immunoprecipitated with the HMGA1 protein and promoted the tumorigenicity of endometrial carcinoma *in vitro* and *in vivo* (48). Lu et al. (49) explored the role of LINC00665 in gemcitabine resistance of cholangiocarcinoma and found that LINC00665 was upregulated in gemcitabine-resistant cells. High LINC00665 expression was positively correlated to advanced TNM stage, lymph node/distant metastasis, and poor prognosis. Assays *in vitro* and *in vivo* indicated that LINC00665 increased the gemcitabine tolerance of cholangiocarcinoma cells by regulating EMT, stemless properties, and the miR-424-5p/BCL9L axis. Through extracting the RNAs from acute myeloid leukemia (AML) or normal bone marrow tissues and cells, an RT-qPCR assay displayed that LINC00665 was upregulated in AML tissues and cell lines. LINC00665 could accelerate the progression of AML by regulating the miR-4458/DOCK1 axis (54). LINC00665 was also found to be upregulated in T-cell acute lymphoblastic leukemia (T-ALL) and could promote T-ALL progression through the miR-101/PI3K/Akt pathway (55).

## Conclusion and Future Perspectives

Mounting evidence has indicated that lncRNAs were dysregulated in human cancers and act as critical regulators in tumorigenesis and tumor progression. Although lncRNA was known as a kind of non-coding RNA, several studies have reported that it has the capacity to code small proteins or micropeptides. LncRNA LOC90024 was found to encode a splicing regulatory small 130-amino acid protein, which could promote the tumorigenesis and progression of CRC (64). In BCa, LINC00665 could encode a biologically active micropeptide CIP2A-BP. However, whether LINC00665 could encode micropeptides in other kinds of cancers remains unclear and needs further exploration. The roles played by lncRNAs differ depending on the subcellular location. LINC00665 was found to be mainly located in the cytoplasm in BCa, osteosarcoma, CRC, glioma, lung cancer, and OC, thus participating in biological regulation through ceRNA, STAU1-mediated mRNA degradation, interfering with RNA-binding proteins, and so on. In PCa, LINC00665 was mainly expressed in the nucleus and may function at the transcriptional level. The subcellular location of LINC00665 in HCC, GC, cervical cancer, and melanoma is still unclear. The present findings showed that LINC00665 was highly expressed in most cancers and functioned as an oncogene in cell proliferation, migration, invasion, and apoptosis. However, the expression status and specific roles of LINC00665 in esophagus cancer, pancreatic cancer, and so on are unknown, and its expression level in glioma is controversial. Further studies should enroll a larger cohort of clinical samples to improve the reliability of studies and focus more on exploring the precise biological regulatory mechanisms of LINC00665.

## Author Contributions

YL and CZ designed the study. CZ, S-NX, KL, and J-HC helped with data processing and reference collection. CZ, J-HC, and QL prepared the figures and tables. All authors participated in revising the final manuscript and approved it for publication.

## Funding

This work was supported by Wu Jieping Medical Foundation (No. 320.6750.19028) and PhD Start-up Fund of Henan Cancer Hospital.

## Conflict of Interest

The authors declare that the research was conducted in the absence of any commercial or financial relationships that could be construed as a potential conflict of interest.

## Publisher’s Note

All claims expressed in this article are solely those of the authors and do not necessarily represent those of their affiliated organizations, or those of the publisher, the editors and the reviewers. Any product that may be evaluated in this article, or claim that may be made by its manufacturer, is not guaranteed or endorsed by the publisher.
